# Evaluating Recalibrating AI Models for Breast Cancer Diagnosis in a New Context: Insights from Transfer Learning, Image Enhancement and High-Quality Training Data Integration

**DOI:** 10.3390/cancers16020322

**Published:** 2024-01-11

**Authors:** Zhengqiang Jiang, Ziba Gandomkar, Phuong Dung (Yun) Trieu, Seyedamir Tavakoli Taba, Melissa L. Barron, Peyman Obeidy, Sarah J. Lewis

**Affiliations:** 1 Discipline of Medical Imaging Science, School of Health Sciences, Faculty of Medicine and Health, The University of Sydney, Sydney 2006, Australia; ziba.gandomkar@sydney.edu.au (Z.G.); phuong.trieu@sydney.edu.au (P.D.T.); amir.tavakoli@sydney.edu.au (S.T.T.); melissa.robinson@sydney.edu.au (M.L.B.); peyman.obeidy@sydney.edu.au (P.O.); 2 School of Health Sciences, Western Sydney University, Campbelltown 2560, Australia

**Keywords:** artificial intelligence, deep learning, radiologists’ concordance, image enhancement, mammography, saliency maps, transfer learning

## Abstract

**Simple Summary:**

Breast cancer is one of the leading causes of cancer-related death in women. The early detection of breast cancer with screening mammograms plays a pivotal role in reducing mortality rates. Although population-based double-reading screening mammograms have reduced mortality by over 31% in women with breast cancer in Europe, continuing this program is difficult due to the shortage of radiologists. Artificial intelligence (AI) is an emerging technology that has provided promising results in medical imaging for disease detection. This study investigates the performance of AI models on an Australian mammographic database, demonstrating how transfer learning from a USA mammographic database to an Australian one, contrast enhancement on mammographic images and the quality of training data according to radiologists’ concordance can improve breast cancer diagnosis. Our proposed methodology offers a more efficacious approach for AI to contribute to radiologists’ decision making when interpreting mammography images.

**Abstract:**

This paper investigates the adaptability of four state-of-the-art artificial intelligence (AI) models to the Australian mammographic context through transfer learning, explores the impact of image enhancement on model performance and analyses the relationship between AI outputs and histopathological features for clinical relevance and accuracy assessment. A total of 1712 screening mammograms (*n* = 856 cancer cases and *n* = 856 matched normal cases) were used in this study. The 856 cases with cancer lesions were annotated by two expert radiologists and the level of concordance between their annotations was used to establish two sets: a ‘high-concordances subset’ with 99% agreement of cancer location and an ‘entire dataset’ with all cases included. The area under the receiver operating characteristic curve (AUC) was used to evaluate the performance of Globally aware Multiple Instance Classifier (GMIC), Global-Local Activation Maps (GLAM), I&H and End2End AI models, both in the pretrained and transfer learning modes, with and without applying the Contrast Limited Adaptive Histogram Equalization (CLAHE) algorithm. The four AI models with and without transfer learning in the high-concordance subset outperformed those in the entire dataset. Applying the CLAHE algorithm to mammograms improved the performance of the AI models. In the high-concordance subset with the transfer learning and CLAHE algorithm applied, the AUC of the GMIC model was highest (0.912), followed by the GLAM model (0.909), I&H (0.893) and End2End (0.875). There were significant differences (*p* < 0.05) in the performances of the four AI models between the high-concordance subset and the entire dataset. The AI models demonstrated significant differences in malignancy probability concerning different tumour size categories in mammograms. The performance of AI models was affected by several factors such as concordance classification, image enhancement and transfer learning. Mammograms with a strong concordance with radiologists’ annotations, applying image enhancement and transfer learning could enhance the accuracy of AI models.

## 1. Introduction

Breast cancer had the highest incidence among all types of solid cancers among women worldwide in 2020, leading to the highest mortality [1]. To reduce mortality, mammography was introduced for breast screening in many countries in the early 2000s. Mammography remains the most common imaging technique for breast cancer diagnosis in most countries, and a standard screening mammogram consists of X-ray imaging with two views on each breast in the mediolateral oblique (MLO) and craniocaudal (CC) projection. Mammographic images in these two views are interpreted by radiologists and other readers to determine whether the screening case is negative for breast cancer or the woman needs to be recalled for further imaging and/or testing. The mortality for women with breast cancer in European populations has reduced by over 31% as attributed to population-based programs using mammography [2]. Women diagnosed with abnormal mammograms are recommended for further testing, which can include additional images or biopsy. Over 60% of these biopsies are diagnosed as cancer-free [3].

Although the sensitivity (>86%) and specificity (>96%) [4] of screening mammography to detect breast cancer for women with almost entirely fatty breasts is relatively high, a major challenge in mammography screening involves women with dense breasts, as breast cancer can be masked by glandular tissue. Tissue superposition occurs in mammography when there are overlapping layers of breast tissue that can obscure small or subtle abnormalities, making it difficult for radiologists to accurately interpret the images [5]. This issue has been partially mitigated by digital breast tomosynthesis (DBT), which is an advanced mammographic technology that captures three-dimensional images of the breast, allowing for a more detailed and layered view of breast tissue. However, the larger volume of images generated by DBT necessitates more time for both image acquisition and interpretation [6].

Over the past decade, artificial intelligence (AI) has garnered extensive attention in medical imaging for its promising advancements in the diagnostic accuracy of interpretative tasks related to various organs like the brain, liver, breast and lung [7,8,9,10,11,12,13,14,15,16,17,18,19]. Particularly, deep learning methods applied to diagnose breast cancer through mammographic images have captivated extensive interest [10,12,16,17]. The effective training of AI models for clinical application demands a vast amount of data containing precise lesion locations. However, the acquisition of these extensive sets of images with lesion locations significantly increases the workload for radiologists and physicians. To mitigate some of these workload challenges, transfer learning [20], involving the use of pretrained AI models in different settings, has emerged as a potential solution.

Breast screening with AI models can assist radiologists in interpreting mammograms, especially in distinguishing between normal and abnormal cases [21]. The Globally-aware Multiple Instance Classifier (GMIC) [16] AI model was designed to classify mammographic cases as benign or malignant. Furthermore, the Global-Local Activation Maps (GLAM) [17] AI model extended GMIC to classify mammographic cases as benign or malignant by generating multiple-scale saliency maps. The I&H AI model [10] used deep neural networks to assist radiologists in interpreting screening mammograms. The End2End AI model [12] demonstrated a method of breast screening on mammograms using deep neural networks. All these four AI models used residual networks (ResNet) architecture [22] in the training and testing processes. For completeness of the paper, a detailed review of these methods is given in the methods section.

This paper investigates the performance of these four publicly available, state-of-the-art AI models, GMIC, GLAM, I&H and End2End, on a screening mammographic database of Australian women. This study’s primary goals include

(1) Comparing the performance of these models on an Australian dataset, which differs from their original training data (both in terms of population characteristics and the types of mammography machines (vendors) used), highlighting the influence of dataset variations on predictions.
(2) Investigating the potential improvement of model performance through transfer learning and, hence, the value of tailoring the AI models for other nationalities’ contexts.
(3) Examining the impact of image enhancement techniques on model predictions to assess their potential to enhance diagnostic accuracy.
(4) Exploring the association between the AI models’ malignancy probability outputs and histopathological features, offering insights into the models’ predictive accuracy and potential clinical relevance, aiding further treatment/triaging decision making.

## 2. Materials and Methods

**Figure 1:**
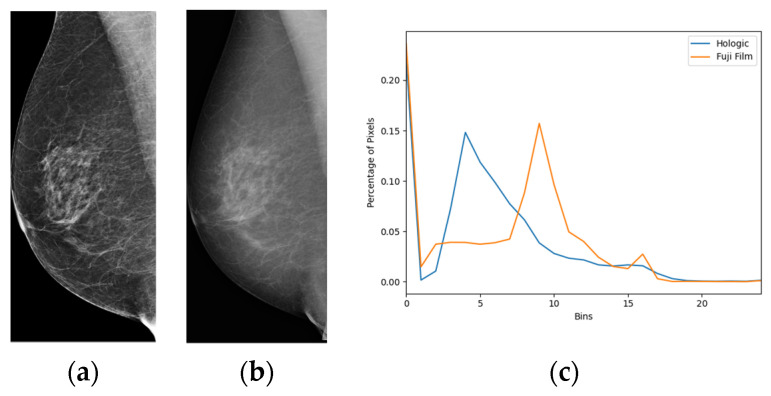
Histogram comparison of mammographic images from different vendors. (**a**) An image from Hologic. (**b**) An image from Fuji Film. (**c**) Histogram comparison of these two images.

Four state-of-the-art AI models involving deep neural networks were used to test an Australian mammographic database. Transfer learning of the four pretrained AI models was conducted on the database to update these AI models. Since the images in our dataset were obtained from different vendors, they exhibited significantly different histograms and dynamic ranges. Figure 1 shows the comparison of two histograms from mammographic images from Hologic (Figure 1a) and Fuji Film (Figure 1b). The bin number of the histograms was set as 25. These two histograms (Figure 1c) are statistically significantly different (*p*-value < 0.001).

**Figure 2:**
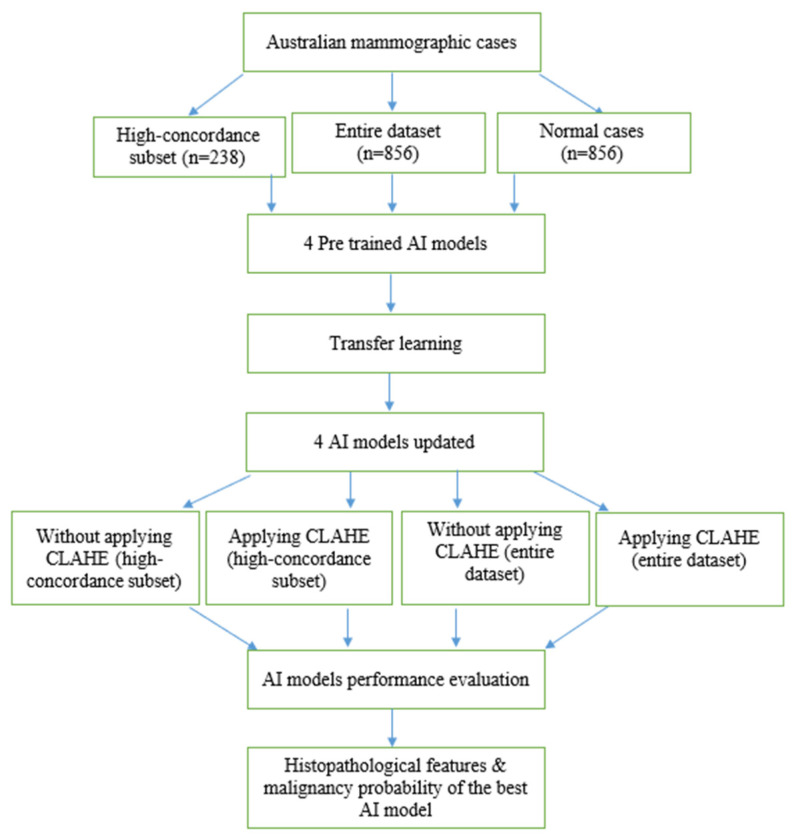
Methodology flow chart.

Therefore, we applied the Contrast Limited Adaptive Histogram Equalization (CLAHE) algorithm [23] to enhance the contrast of mammographic cases and evaluated its impact on the performance of AI models. The receiver operating characteristic curve (ROC) and the area under the ROC curve (AUC) metrics were used to evaluate the performance of the four AI models in different scenarios. Histopathological features were analysed with the malignancy probabilities of mammographic cases to provide the best AI model in terms of AUC values. Our method consisted of several steps, as illustrated in Figure 2.

### 2.1. Data Acquisition

**Figure 3:**
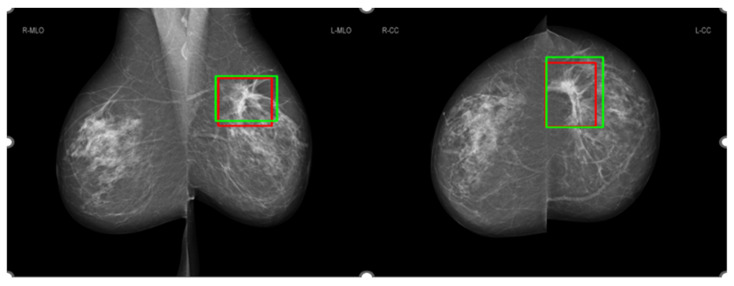
Annotations from Radiologist A in red and Radiologist B in green.

**Figure 4:**
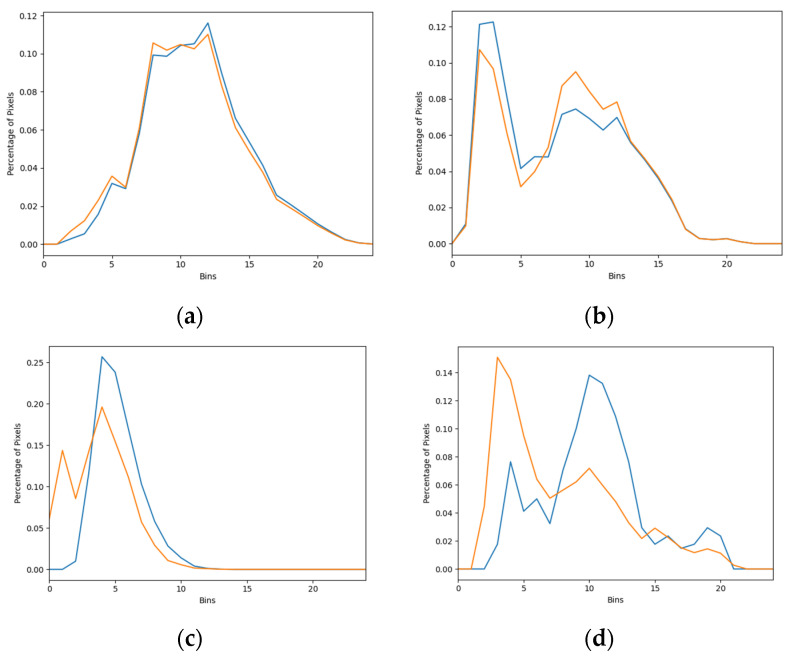
Histograms of annotations from Radiologists A in blue and B in orange in four concordance levels. (**a**) Almost perfect, (**b**) substantial, (**c**) moderate, (**d**) poor.

After ethics approval from the University of Sydney, we used screening mammograms collected from the Australian mammographic database Lifepool to assess the performance of the four AI models. The Lifepool database consists of 1712 mammographic cases (856 normal cases and 856 malignant cases). Each malignant case was confirmed by the reports of follow-up biopsies. Each case had four mammographic views: right MLO, left MLO, right CC and left CC views. Mammograms were acquired from mammography machines manufactured by five different vendors, including Fuji Film (32% of cases), Konica (4% of cases), Siemens (34% of cases), Hologic (19%) and Sectra (11% of cases). Each case was annotated by two radiologists and recorded as box regions on the mammographic images. Figure 3 shows an example of the annotations of two radiologists on a mammographic case, with red boxes from Radiologist A and green boxes from Radiologist B. Concordance levels were constructed by analysing Lin’s concordance correlation coefficient (CCC) [24] between the annotations of two radiologists on mammograms according to McBride’s interpretation guide [25]. Lin’s CCC was computed based on the corners of two overlapped boxes of annotations on the same mammographic image. The Intersection over Union [26] metric was used to determine whether two boxes overlapped or not, with a value greater than 0 indicating the overlapping of two boxes. Mammographic images were classified as four concordance levels: ‘almost perfect’ at >0.99 (238 cases), ‘substantial’ at <0.95 to 0.95 (222 cases), ‘moderate’ at 0.95 to 0.90 (202 cases) and ‘poor’ at <0.90 (194 cases). Figure 4 shows the histograms of annotations from the two radiologists in four concordance levels. A correlation metric [27] was used to compare the two histograms. The correlation metric is in the range [0, 1]. A larger correlation value indicates more overlap between the two histograms. For the almost perfect, substantial, moderate and poor levels in Figure 4a–d, the correlation value was 0.996, 0.962, 0.811 and 0.480, respectively. A larger correlation value with the higher concordance level indicated that the thresholds were suitable for different concordance levels.

The training and testing mammographic cases of our database had an equal representation of breast density. Two image sets were developed: the first subset included cases rated with ‘almost perfect’ agreement between radiologists (termed ‘high-concordance subset’ in this paper), and the second dataset included all cases that have been marked with cancers with ‘no concordance threshold’ applied (termed ‘entire dataset’ in this paper).

### 2.2. AI Models

The GLAM, GMIC, I&H and End2End models were evaluated in this study. These four models were selected as each model provided promising results in diagnosing cancers on mammographic images with high AUC values. The GMIC model combined the global and local context in the decision-making process [16]. To obtain additional details of the local context, the GLAM incorporated zoom functionality for the local context, similar to the approach taken by radiologists interpreting mammographic images [17]. To mimic radiologists interpreting mammographic images from different views, I&H fused each model trained on each view for the decision-making process [10], as sometimes a mammographic image from a single view is not enough to determine whether the mammographic image shows cancer. Instead of searching cancer signs in a direction from the global to the local on a mammographic image like GMIC and GLAM, End2End trained a local classifier and then expanded to a global classifier to determine whether the mammographic images showed signs of cancer. Although the AUC values reported previously for GMIC, GLAM, I&H and End2End using their original mammography databases were 0.909, 0.882, 0.895 and 0.88, respectively, these AI models have reportedly provided relatively low AUC values on other mammographic databases from different ethnicities and manufacturers [28].

#### 2.2.1. Globally-Aware Multiple Instance Classifier (GMIC)

The GMIC first learned the global feature map of a mammographic image using a ResNet-22 network [25]. The global feature map was convolved with a 1 × 1 filter and Sigmoid operation to generate a malignant map. The value of each pixel in the global feature map was [0, 1], which indicated the presence of malignancy. The feature map was then scanned to obtain non-overlapping *K* patches with the largest total intensity inside the patches. As suggested in the original paper, *K* was set as 3. Local features of patches were extracted using a ResNet-34 network and then combined with a gated attention network for computing the weights of features. The final step combined the malignant map and local feature with the weighted representation of all patches to predict malignancy probability. All the mammographic images for GMIC models were resized to a resolution of 1920 × 2944 pixels using bilinear interpolation [29]. For the GMIC model, the source codes are publicly available on GitHub at https://github.com/nyukat/GMIC.git (accessed on 2 November 2022).

#### 2.2.2. Global-Local Activation Maps (GLAM)

The GLAM learned the global saliency map of a mammographic image using a convolutional neural network (CNN). To capture different sizes of malignancy, the global saliency map was generated at different scales. The second stage generated a set of patches from the feature map based on the local maximum of average intensity. In the last stage, each image patch was applied to a ResNet-34 network [22] to extract the local feature map, which was then assigned to the corresponding mammographic image. All feature maps of local patches were combined with the global feature map to predict the probability of malignancy on a mammographic image using the binary cross-entropy function. All the mammographic images for GLAM models were also resized to a resolution of 1920 × 2944 pixels. For the GLAM model, the source codes are publicly available on GitHub at https://github.com/nyukat/GLAM.git (accessed on 2 November 2022).

#### 2.2.3. I&H

I&H trained AI models based on MLO and CC views on each breast and concatenated representations from four views to predict the probability of malignancy in each mammographic image. ResNet-22 was used for model training in a mammographic image of each view. The mammographic images in CC view for the I&H model were resized to 2677 × 1942 and 2974 × 1748 in MLO view. For this model, we used the source codes published by the authors on GitHub at https://github.com/nyukat/breast_cancer_classifier.git (accessed on 2 November 2022).

#### 2.2.4. End2End

End2End converted a patch classifier to a whole mammographic image classifier by adding heatmaps and convolutional layers on the top of the neural network. These convolutions used two visual geometry group (VGG) [30] blocks with 3 × 3 convolutions and batch normalization. All the mammographic images for End2End were resized to a resolution of 1152 × 896 pixels. For this model, we used the source codes published by the authors on GitHub at https://github.com/lishen/end2end-all-conv.git (accessed on 2 November 2022).

### 2.3. Image Enhancement

**Figure 5:**
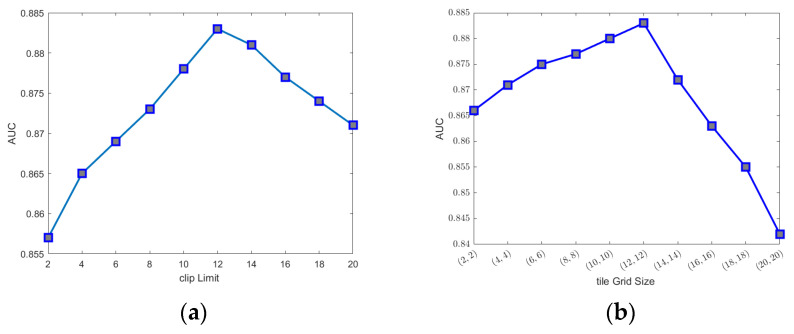
AUC values of the GMIC on Lifepool versus different values of the clip limit and tile grid size. (**a**) The tile grid size = (8, 8). (**b**) The clip limit = 12.

Image enhancement techniques can be helpful to optimize the contrast of mammographic images and one example is from Min et al. [31], where the study presented pseudo-colour mammogram generation to enhance mass-like features in mammographic images. In this study, we used the CLAHE [23] algorithm to enhance mammographic images because it is fast and produces promising contrast enhancement. The CLAHE algorithm first divided an image into un-overlapped tiles. In the second stage, it conducted histogram equalization for each tile. The histogram equalization used a predefined clip limit to redistribute the bins and then map to an improved tile. The last stage combined each improved tile to generate an enhanced image using bilinear interpolation. We have conducted preliminary experiments on the mammographic database to empirically determine the values of parameters of the CLAHE algorithm. Figure 5 depicts the AUC of the GMIC on Lifepool versus the two parameters. In Figure 5a, the tile grid size was fixed to (8, 8); in Figure 5b, the clip limit was fixed to 12. In both the plots, we can see that AUC increased with the clip limit up to 12 and tile grid size (8, 8) and then decreased afterward. Therefore, the clip limit was set to 12 and the tile grid size was set to (8, 8) in all the experiments reported in this paper.

### 2.4. Transfer Learning

Transfer learning of the four AI models was conducted on the Lifepool database, including 856 cancer cases and 856 normal cases. The resolutions of most original images from Lifepool DICOM data are 4728 × 5928, but the images for training AI models are in PNG format with much smaller resolutions (e.g., GMIC and GLAM with 1920 × 2944). All DICOM images were downsampled to match the resolution of the input images for the models and converted to PNG format to reduce the computational time of the training process. We conducted a four-fold cross-validation to train and test the four AI models on the database with transfer learning. The training set was further split into training and validation sets to refine the stopping criteria. This step involved an iterative process, assessing the AI models’ accuracy in the current epoch against the previous one. The training concluded when the validation process callback showed no improvement in model accuracy, typically after a patience threshold of 3 epochs had been reached. 

The transfer learning of each AI model was optimized using the Adam algorithm [32]. The loss function used the binary cross-entropy. As suggested in the original studies, the learning rates for the GMIC, GLAM and I&H were set as 10^−5^ and End2End was set as 10^−4^, respectively. For an equitable comparison of performance between transfer learning models and pretrained models, the transfer learning approach employed the ResNet-22 network for the global module and the ResNet-34 network for the local module. These are the same networks utilized by the pretrained GMIC and GLAM models. Additionally, I&H utilized the ResNet-22 network as its pretrained model, while End2End employed the VGG network as its pretrained model.

### 2.5. Evaluation Metrics

The performance of four AI models in the classification of malignancy on mammographic images was evaluated using sensitivity, specificity and the area under the receiver operating characteristic curve (AUC). An ANOVA test was conducted for each AI model between the two image sets, with the corresponding *p*-values as shown in the Results section. The DeLong test [33] was also used to compare AUC values from the four AI models between the original and transfer learning modes on the high-concordances subset and entire dataset. The Youden index [34] was used to assess objectively the ROC curves of the four AI models, both in original and transfer learning modes and with and without contrast enhancement. A threshold of statistical significance was set as 0.05. Bonferroni correction was used to adjust for multiple comparisons. 

### 2.6. Association between the Malignancy Probability from the AI and Histopathological Features

We also employed the Kruskal–Wallis U-test to investigate potential differences in malignancy probability as predicted by the top-performing AI model across distinct categories based on pathology reports. We considered pathological factors including estrogen receptor (ER), progesterone receptor (PR), breast cancer grade, human epidermal growth factor receptor 2 (Her2) and the differentiation between ductal carcinoma in situ (DCIS) and invasive cancer. Additionally, an analysis was conducted based on the size of the cancers, with tumours classified into four groups (mm): (0.999, 10.0], (10.0, 15.0], (15.0, 25.0] and (25.0, 150.0] intervals. The Kruskal–Wallis U-test was utilized to assess the statistical significance of differences among these groups. 

## 3. Results

### 3.1. The Performances of Four AI Models

In the pretrained stage, GMIC obtained a significantly higher AUC score in both the high-concordance subset and the entire dataset in original (0.865 and 0.824) and contrast-enhanced (0.870 and 0.836) modes, followed by the GLAM, I&H and then End2End models (Table 1). There were significant differences (*p* < 0.05) (Table 1) in the performances of these models between the two datasets. The AUC values of the four AI models were higher when the CLAHE image enhancement algorithm was applied, in comparison with the original mammograms (Table 1) (e.g., 0.870 for GMIC + CLAHE vs. 0.865 for GMIC only in the high-concordance subset, and 0.836 for GMIC + CLAHE vs. 0.824 for GMIC only in the entire dataset).

In the transfer learning stage, the highest AUC score was found with the GMIC for both the high-concordance subset and the entire dataset (0.910 and 0.883) and again with the contrast-enhanced (0.912 and 0.889) mode compared with the values generated by the GLAM, I&H and then End2End’s models without contrast enhancement (Table 1). Significantly higher AUC scores were also reported in the subset than in the entire dataset across four models with and without contrast enhancement (*p* < 0.05) (Table 1). There was an improvement in the AUC values of the four transfer learning AI models on the contrast-enhanced mammograms compared with the original mammograms in both datasets, as shown in this table (e.g., 0.912 for GMIC + CLAHE vs. 0.910 for GMIC only in the high-concordance subset, and 0.889 for GMIC + CLAHE vs. 0.883 for GMIC only in the entire dataset).

Table 2 shows the *p*-values of the DeLong test for the comparison of AUC values from four AI models between the original and transfer learning modes with and without the CLAHE image enhancement algorithm on both the entire dataset and the high-concordance data subset. From this table, we can see that the improvement in the AUC values of all four AI models between the original and transfer learning modes was statistically significant on both datasets.

**Figure 6:**
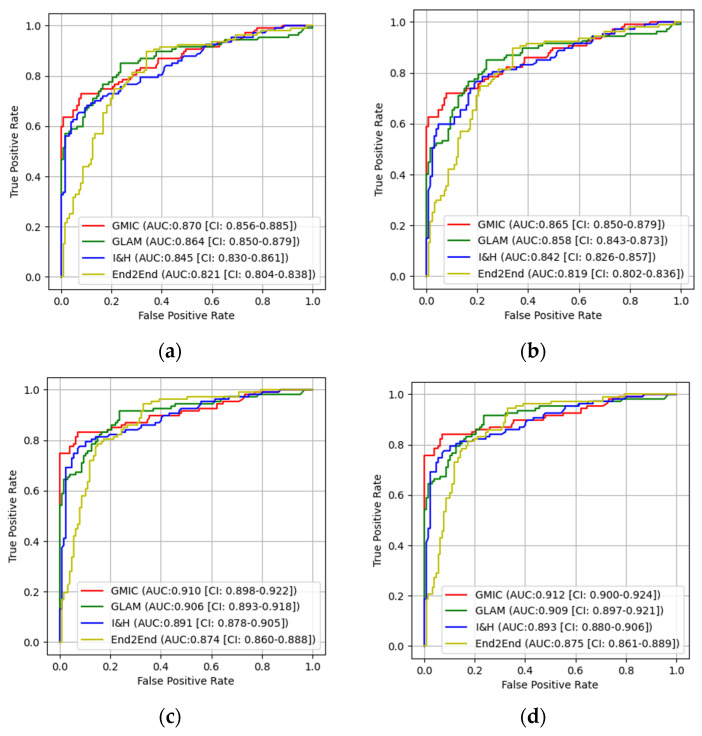
The receiver operating characteristic curves (ROC) of the four AI models on high-concordance subset. (**a**) ROC curves of the AI models on original mammographic images; (**b**) the ROC curves of the AI models on enhanced mammographic images; (**c**) the ROC curves of the AI transfer learning models on original mammographic images; (**d**) the ROC curves of the AI transfer learning models on enhanced mammographic images.

**Figure 7:**
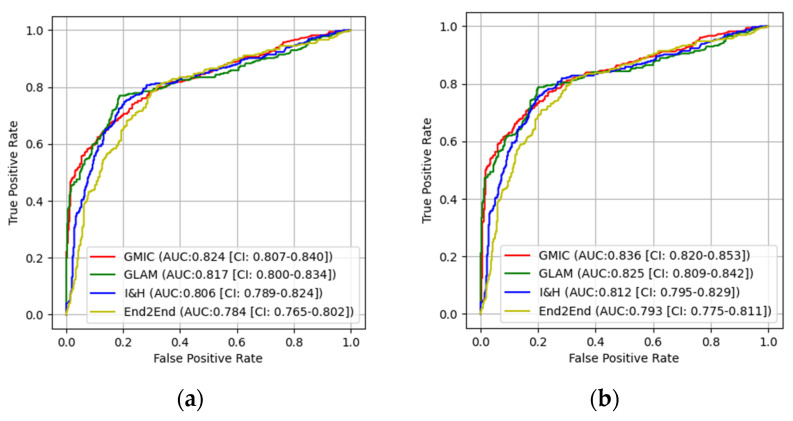

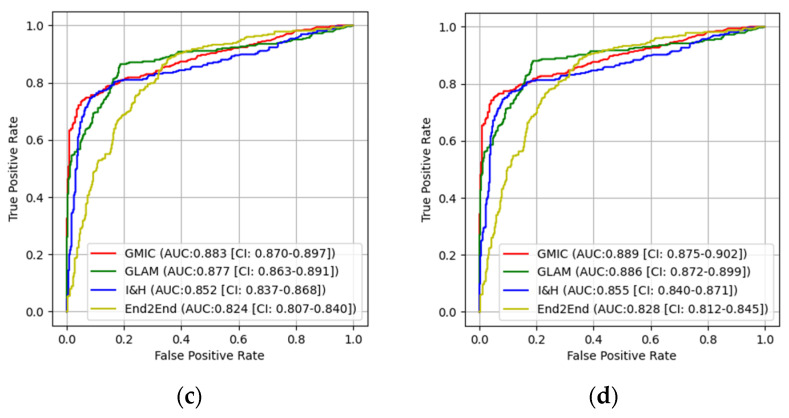
The receiver operating characteristic curves (ROC) of the four AI models on entire dataset. (**a**) ROC curves of the AI models on original mammographic images; (**b**) the ROC curves of the AI models on enhanced mammographic images; (**c**) the ROC curves of the AI transfer learning models on original mammographic images; (**d**) the ROC curves of the AI transfer learning models on enhanced mammographic images.

Figure 6 and Figure 7 show the comparison of ROC curves of the four AI models with and without transfer learning on the high-concordance subset and entire dataset, respectively. The ROC curves in these figures show a clear improvement in performance among the four AI models with transfer learning (see Figure 6 and Figure 7a,c) and CLAHE contrast enhancement (see Figure 6 and Figure 7b,d). Confidence intervals for the four AI models on the high-concordance subset are shown in the legend of each subfigure.

Figure 6 and Figure 7 also illustrate that the receiver operating characteristic (ROC) curves of the four AI models, both with and without transfer learning and with and without contrast enhancement, exhibited superior performance in terms of the Youden index (e.g., 0.650 for GMIC + CLAHE vs. 0.641 for GMIC only in Figure 6a without transfer learning, 0.770 for GMIC + CLAHE vs. 0.761 for GMIC only in Figure 6d with transfer learning) in the high-concordance subset compared to the entire dataset (e.g., 0.548 for GMIC + CLAHE vs. 0.520 for GMIC only in Figure 7a without transfer learning, 0.698 for GMIC + CLAHE vs. 0.678 for GMIC only in Figure 7d with transfer learning). The ROC curves of the four AI transfer learning models showed more improvement on two datasets than those of the four pretrained AI models (e.g., Figure 6a and Figure 7a vs. Figure 6c and Figure 7c).

### 3.2. Pairwise Comparisons of Four AI Models

We conducted pair-wise comparisons among the models in various scenarios to explore if the differences in the performances were significant. In each scenario, six comparisons were made and the *p*-values were adjusted using Bonferroni correction. As shown in Table 3, the differences were more significant when models were recalibrated using transfer learning. This highlights the need for transfer learning to leverage the maximum added benefit of the model. The GMIC and GLAM models were not significantly different in the entire dataset because both models have a similar architecture of networks and GLAM was an extended work of GMIC. 

The I&H and GMIC or GLAM models were not significantly different when using the original or contrast-enhanced images in the entire dataset, but significant differences were observed when transfer learning models were used. The GMIC and End2End models were significantly different in both the high-concordance subset and the entire dataset due to the different deep neural network architectures of the two models (one with ResNet and the other with VGG). 

### 3.3. Comparison of Salience Maps on Original and Locally Enhanced Mammographic Images

**Figure 8:**
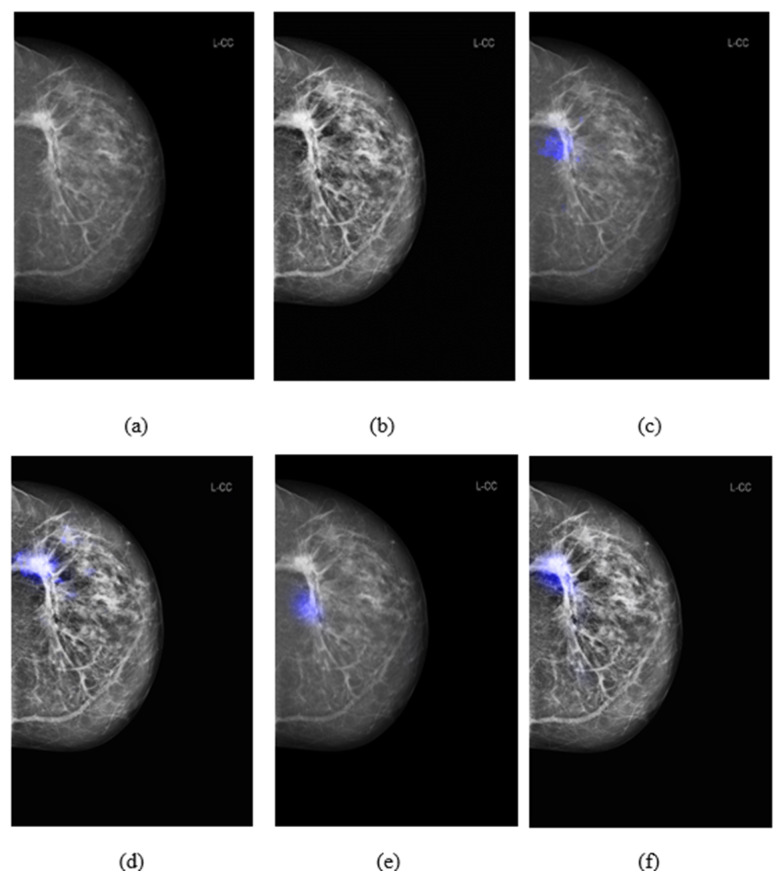
Comparison of saliency map from GLAM and GMIC on an original mammographic image with and without applying Contrast Limited Adaptive Histogram Equalization (CLAHE) algorithm. (**a**) Original mammogram in CC view; (**b**) enhanced mammogram using CLAHE algorithm; (**c**) saliency maps on the original mammogram using GLAM; (**d**) saliency maps on the enhanced mammogram using GLAM; (**e**) saliency maps on the original mammogram using GMIC; (**f**) saliency maps on the enhanced mammogram using GMIC.

Figure 8 shows the comparison of saliency maps generated from GLAM and GMIC on both an original mammographic image and with the applied CLAHE algorithm. The annotations of two radiologists on the same mammographic case are shown in the left CC view in Figure 3. From Figure 8, we can see that the saliency maps of GLAM (see Figure 8c) and GMIC (see Figure 8e) from original mammographic images deviated from the centroid of the radiologists’ annotations and occupied a smaller area of the annotations. However, the saliency maps of the two AI models from the contrast-enhanced image (see Figure 8d,f) aligned with the centroid of radiologists’ annotations and occupied a larger area of the annotations.

### 3.4. Association between the Malignancy Probability from the AI and Histopathological Features

The outcomes of the Kruskal–Wallis tests, assessing the significance of differences in malignancy probability predicted by the highest-performing AI model (GMIC) across various pathological factors, revealed nonsignificant findings. The comparison based on ER, PR and Her2 status yielded *p*-values of 0.342, 0.414 and 0.179, respectively. The examination of breast cancer grade resulted in a *p*-value of 0.169. Additionally, the differentiation between DCIS (503 cases) and invasive cancer (312 cases) exhibited a nonsignificant *p*-value of 0.152.

**Figure 9:**
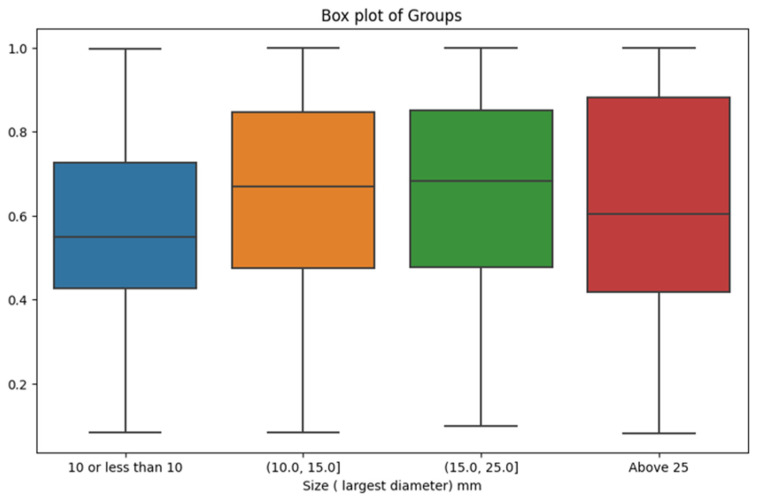
Box plot depicting the relationship between tumour size categories and corresponding malignancy probability scores from the highest-performing AI model.

However, when investigating the impact of tumour size categories on malignancy probability, the results were statistically significant. There were 337 cases with tumour size in (0, 10.0 mm], 174 cases in (10.0, 15.0 mm], 179 cases in (15.0, 25.0 mm] and 166 cases above 25 mm. The analysis yielded a *p*-value of 0.0002, indicating that the distinct size groups indeed manifested significant differences in malignancy probability provided by the AI model. As shown in Figure 9, the most prominent difference was observed between the first size category (i.e., lesions with a size of 10 mm or less) with the lowest malignancy probability scores compared with the other size intervals.

## 4. Discussion

In previous studies, the mammograms for training and testing the GLAM, GMIC and I&H were conducted with the New York University Breast Cancer screening database [35], which included examinations from two manufacturers: Siemens and Hologic. The training and testing data for End2End were film-screen (FS) mammographic images from the Digital Database for Screening Mammography (DDSM) [36]. Our dataset included digital mammographic images collected from a wider range of vendors such as Sectra, Fuji, Siemens, Hologic, GE Healthcare and Philips Healthcare. The mammographic images from the NYU and DDSM databases were obtained in the USA, whilst our dataset was obtained in Australia and could represent different populations, with the majority ethnicity group of our database unlikely to be matched with the USA databases. Previous research has shown an 8% difference in the AUC of an AI model on US screening mammograms and UK screening mammograms [11].

Our results showed that transfer learning improved the performance of the four AI models in detecting cancer lesions on digital screening mammograms. As shown in Table 1, the AUC of the transfer learning GMIC model increased from 0.865 for the pretrained model to 0.910 in the high-concordance subset and from 0.824 to 0.883 in the entire dataset. Similar results were also found for GLAM, I&H and End2End. This indicates that transfer learning of the four models was influenced by the quality of the concordance levels, indicating that high-quality data together with undertaking transfer learning were both important factors for training an effective AI model.

Applying image enhancement via the CLAHE algorithm to our image set improved the performance of the AI models in detecting cancer lesions on screening mammograms. The AUC values of the four AI models were greater than those without enhanced mammographic images. Other image enhancements such as pseudo-colour mammogram generation and the local histogram equalization algorithm [37] may also improve the AUC performance of AI models, and this could be a direction for future work.

We also explored the prediction of malignancy probability by the GMIC as the highest-performing model across various pathological factors. Despite nonsignificant differences observed in the context of ER status, PR status, breast cancer grade, Her2 status and the distinction between DCIS and invasive cancer, our investigation showed an association between tumour sizes and AI’s output. The exploration of tumour size categories revealed a highly significant variance in malignancy probability, with the most notable contrast emerging between the initial size category (tumours measuring 10 mm or less) and the subsequent size intervals. This finding highlighted the AI’s potential limitation in confidently annotating malignancy in cases of small tumours and that radiologists should be mindful of the association between lower AI-assigned probability scores and smaller tumour sizes. This insight reinforced the need for a nuanced understanding of AI results and their contexts in clinical practice.

To evaluate the four models, we investigated the performance of the AI models from the point of view of malignancy detection or reporting as a normal case. We did not include any cases with benign lesions in our Australian database, so the results cannot comment on the models’ ability to identify cases with benign features, and this may include cases that were benign but more challenging to AI and human readers. With transfer learning and contrast enhancement application, the AUC of GMIC with CLAHE in the high-concordance subset was 0.912, which is also the best model of the four AI models in this study. It is imperative to engage in transfer learning when mammograms are gathered from distinct populations or various vendors as the performance of AI models can be influenced by the specific vendor or population, necessitating adaptation for optimal results.

## 5. Conclusions

In this paper, we presented the performance of four publicly available AI models for breast cancer detection in different situations such as concordance classification of annotations in the input data, the incorporation of contrast enhancement and the application of transfer learning. The results showed that when tested on the high-concordance subset, these four AI models outperformed their performance on the entire dataset. Improvements in the performance of AI models were observed through the application of contrast enhancement to mammograms and the utilization of transfer learning. In addition, the AI models’ malignancy probability scores were notably influenced by the sizes of the tumours visible in the mammograms. Applying concordance classifications, transfer learning and contrast enhancement of mammograms to AI models is likely to provide an effective method for AI assisting decision making when radiologists interpret mammographic images.

## Figures and Tables

**Table 1:** Performance comparison of four AI models with and without CLAHE image enhancement algorithm on both entire dataset (AUC_Entire_) and the high-concordance data subset (AUC_High_). Two different scenarios were considered: using the original models and using the models recalibrated for our dataset using transfer learning.

	Original			Transfer Learning		
	AUC_Entire_	AUC_High_	*p*-Values	AUC_Entire_	AUC_High_	*p*-Values
GMIC	0.824	0.865	0.0283	0.883	0.91	0.0416
GLAM	0.817	0.858	0.0305	0.877	0.906	0.0359
I&H	0.806	0.842	0.0454	0.852	0.891	0.0257
End2End	0.784	0.819	0.0368	0.824	0.874	0.0162
GMIC + CLAHE	0.836	0.870	0.0137	0.889	0.912	0.0348
GLAM + CLAHE	0.825	0.864	0.0181	0.886	0.909	0.0310
I&H + CLAHE	0.812	0.845	0.0339	0.855	0.893	0.0185
End2End + CLAHE	0.793	0.821	0.0286	0.828	0.875	0.0124

**Table 2:** DeLong test for the AUC values of four AI models between the original and transfer learning modes with and without CLAHE image enhancement algorithm on both entire dataset (AUC_Entire_) and the high-concordance data subset (AUC_High_).

	AUC_Entire_	AUC_High_
GMIC	<0.001	0.0034
GLAM	<0.001	0.0041
I&H	<0.001	0.0002
End2End	0.0093	0.0008
GMIC + CLAHE	<0.001	0.004
GLAM + CLAHE	<0.001	0.0121
I&H + CLAHE	<0.001	0.0032
End2End + CLAHE	0.0219	0.001

**Table 3:** The *p*-values for pair-wise comparison of the models’ output in different scenarios (significant level of 0.0083 was considered after applying Bonferroni adjustment). The *p*-values were adjusted using Bonferroni correction.

Model Images	Without Transferred Learning, Original		Without Transferred Learning, CLAHE		With Transferred Learning, Original		With Transferred Learning, CLAHE	
Dataset	Entire	High	Entire	High	Entire	High	Entire	High
GMIC vs. GLAM	0.0362	0.0624	0.0331	0.0566	0.0193	0.0233	0.0141	0.0215
GMIC vs. I&H	0.0175	0.0387	0.0108	0.0369	0.0076	0.0135	0.0058	0.0121
GMIC vs. End2End	0.0062	0.0078	0.0049	0.0062	0.0027	0.0041	0.0015	0.0030
GLAM vs. I&H	0.0236	0.0294	0.0217	0.0279	0.0061	0.0093	0.0020	0.0075
GLAM vs. End2End	0.0064	0.0186	0.0059	0.017	0.0073	0.0142	0.0057	0.0128
I&H vs. End2End	0.0081	0.0351	0.0025	0.0344	0.0220	0.0327	0.0106	0.0310

## Data Availability

The data supporting this study’s findings are available on request from the corresponding authors.
